# Combined plasma C‐reactive protein, interleukin 6 and YKL‐40 for detection of cancer and prognosis in patients with serious nonspecific symptoms and signs of cancer

**DOI:** 10.1002/cam4.5455

**Published:** 2022-11-28

**Authors:** Alex N. Videmark, Ib J. Christensen, Claus L. Feltoft, Mette Villadsen, Frederikke H. Borg, Barbara M. Jørgensen, Stig E. Bojesen, Caroline Kistorp, Randi Ugleholdt, Julia S. Johansen

**Affiliations:** ^1^ Department of Medicine Copenhagen University Hospital ‐ Herlev and Gentofte Herlev Denmark; ^2^ Department of Gastroenterology Copenhagen University Hospital ‐ Amager and Hvidovre Hvidovre Denmark; ^3^ Department of Clinical Biochemistry Copenhagen University Hospital ‐ Herlev and Gentofte Herlev Denmark; ^4^ Department of Clinical Medicine, Faculty of Health and Medical Sciences University of Copenhagen Copenhagen Denmark; ^5^ Department of Endocrinology Copenhagen University Hospital ‐ Rigshospitalet Copenhagen Denmark; ^6^ Department of Oncology Copenhagen University Hospital ‐ Herlev and Gentofte Herlev Denmark

**Keywords:** biomarker, cancer, CRP, IL‐6, YKL‐40

## Abstract

**Background and methods:**

Inflammation is a hallmark of cancer and its progression. Plasma levels of C‐reactive protein (CRP), interleukin‐6 (IL‐6) and YKL‐40 reflect inflammation, and are elevated in patients with cancer. This study investigated whether plasma CRP, IL‐6 and YKL‐40 had diagnostic value in 753 patients referred with nonspecific signs and symptoms of cancer to a diagnostic outpatient clinic.

**Results:**

In total, 111 patients were diagnosed with cancer within 3 months and 30 after 3 months. CRP, IL‐6 and YKL‐40 were elevated in 44%, 60% and 45% of the cancer patients, and in 15%, 33% and 25% of the patients without cancer. Elevated levels of all three markers were associated with risk of cancer within 3 months: CRP (odds ratio (OR) 4.41, 95% confidence interval (CI) 2.86–6.81), IL‐6 (OR = 2.89, 1.91–4.37) and YKL‐40 (OR = 2.42, 1.59–3.66). Multivariate explorative analyses showed that increasing values were associated with the risk of getting a cancer diagnosis (continuous scale: CRP (OR = 1.28, 1.12–1.47), carcinoembryonic antigen (CEA) (OR = 1.61, 1.41–1.98), CA19‐9 (OR = 1.15, 1.03–1.29), age (OR = 1.29, 1.02–1.63); dichotomized values: CRP (OR = 2.54, 1.39–4.66), CEA (OR = 4.22, 2.13–8.34), age (OR = 1.42, 1.13–1.80)). CRP had the highest diagnostic value (area under the curve = 0.69). Combined high CRP, IL‐6 and YKL‐40 was associated with short overall survival (HR = 3.8, 95% CI 2.5–5.9, *p* < 0.001).

**Conclusion:**

In conclusion, plasma CRP, IL‐6 and YKL‐40 alone or combined cannot be used to identify patients with cancer, but high levels were associated with poor prognosis. CRP may be useful to indicate whether further diagnostic evaluation is needed when patients present with nonspecific signs and symptoms of cancer.

## Graphical abstract

We investigated whether CRP, IL‐6 and YKL‐40 had diagnostic and prognostic value in patients referred with nonspecific signs and symptoms of cancer. Patients with elevated CRP, IL‐6 and YKl‐40 alone or in combination had significantly shorter overall survival.

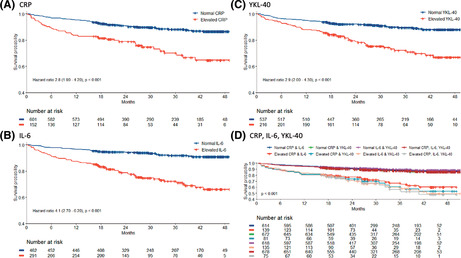

## 1 INTRODUCTION

Cancer is a major global burden, with an estimated 18.1 million new cancer cases and 9.6 million deaths yearly.^1^, ^2^ Early diagnosis is important for the curability of cancers, but the European Society of Medical Oncology (ESMO) and the American Society for Clinical Oncology (ASCO) guidelines do not recommend the few known protein tumour biomarkers for screening of patients suspected of having cancer. Today, only carcinoembryonic antigen (CEA), carbohydrate antigen (CA) 19‐9, cancer antigen (CA) 125 and prostate‐specific antigen (PSA) are used routinely in daily clinical practice in the management of patients with known cancer, but their sensitivity and specificity are too low to be used in screening of patients suspected of having cancer.^3^, ^4^, ^5^, ^6^, ^7^, ^8^, ^9^

Inflammation is one of the hallmarks of cancer,^10^ since it plays an important role in its development and progression.^11^, ^12^, ^13^ The stromal elements in the tumour microenvironment have a dynamic interaction with cancer cells and can influence growth and metastatic potential, and cancer‐associated fibroblasts modulate the extracellular matrix and promote motility, invasion and angiogenesis.^14^, ^15^ The relationship between chronic inflammation, as measured by inflammatory circulating biomarkers including C‐reactive protein (CRP) and interleukin‐6 (IL‐6), and cancer incidence has recently been described in a systematic review and meta‐analysis.^16^

Plasma CRP is the most widely used biomarker of inflammation, although CRP is produced mainly by hepatocytes and not by inflammatory cells.^17^ Other circulating biomarkers of inflammation, like IL‐6 and YKL‐40, are secreted by inflammatory cells, stromal cells and cancer cells.^18^, ^19^, ^20^ IL‐6 stimulates CRP secretion, regulates stromal desmoplasia, promotes tumour‐induced immunosuppression and angiogenesis, inhibits apoptosis, stimulates cancer cell proliferation and facilitates metastasis, including formation of a pro‐metastatic niche in the liver.^17^, ^21^, ^22^ High plasma IL‐6 levels are associated with short overall survival (OS) in patients with inflammatory diseases^19^ and different types of cancer.^23^, ^24^, ^25^, ^26^, ^27^

YKL‐40, also known as chitinase 3‐like 1 protein, stimulates angiogenesis, cell proliferation and differentiation, re‐modulates extracellular matrix, activates Akt signalling, protects against apoptosis and promotes metastases and cancer progression.^20^ In the general population, high levels of plasma YKL‐40 are associated with increased risk of gastrointestinal cancer and death from gastrointestinal cancer.^28^, ^29^, ^30^, ^31^ In patients with various solid tumours, high plasma YKL‐40 is associated with a short OS.^32^

In order to improve the long diagnostic interval, the poor survival of Danish cancer patients and to reduce the time between the first suspicion of cancer and a diagnosis of cancer, a diagnostic fast‐track patient pathway was designed and implemented in 2012 in Denmark for patients presenting with nonspecific symptoms of cancer.^33^, ^34^ Identification of cancer risk‐stratifying biomarkers at first visit might accelerate this process further.

We therefore tested the hypothesis that elevated plasma CRP, IL‐6 and YKL‐40, alone and as a combined biomarker score, could identify groups of patients with nonspecific signs and symptoms of cancer, with high cancer risk and poor survival. We investigated this prospectively in 753 patients included in the Danish MICA study (“New biomarkers in patients referred because of suspected serious illness—are they giving new diagnostic information”).

## 2 MATERIALS AND METHODS

### 2.1 Study design

**FIGURE 1:**
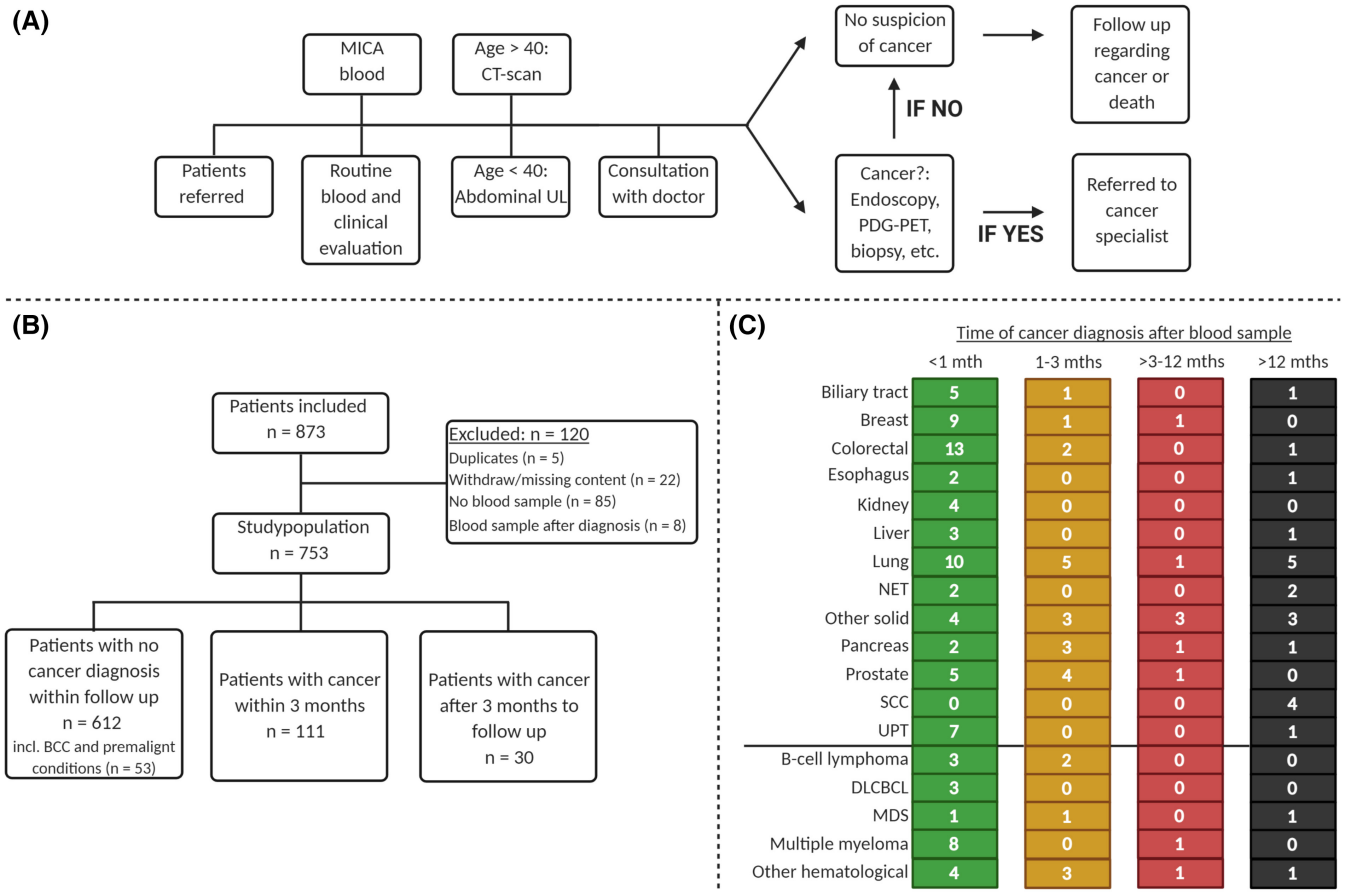
Flowchart showing diagnostic workup of patients referred to the Diagnostic Outpatient Clinic (A) and the inclusion and exclusion of patients to produce the final study group (B). (C) illustrates the number of patients diagnosed with cancer within 1, 1–3, 3–12 months, and after 12 months. DLBCL, diffuse large B‐cell lymphoma; MDS, myelodysplastic syndrome; MGUS, monoclonal gammopathy of unknown significance; SCC, squamous cell carcinoma; UPT, unknown primary tumour. Other haematological: Chronic myeloid leukaemia, Hodgkin's lymphoma, T‐cell lymphoma, Follicular lymphoma, extraosseous plasmacytoma. Other: Gastric cancer, gastrointestinal stromal tumour, granular cell tumour, melanoma and endometrial, head and neck, oesophagus, ophthalmological, and thyroid cancer.

The Danish MICA study was initiated in July 2016 with the aim of conducting translational biomarker research regarding early detection of cancer. The MICA study is an open cohort study including patients (older than 18 years) referred to the diagnostic cancer patient pathway at the Diagnostic Outpatient Clinic at Copenhagen University Hospital—Herlev and Gentofte in the Capital Region of Denmark (Figure 1A). The patients are followed from time of first visit to the Diagnostic Outpatient Clinic to death, emigration or October 20, 2020, whichever came first. Relevant clinical characteristics of the patients are included in the MICA database.

All patients gave written informed consent. The study was performed according to the declaration of Helsinki. The MICA study protocol was approved by the Danish Regional Ethics Committee (H‐7‐2014‐011) and the Danish Data Protection Agency (HEH‐2014‐105; I‐Suite 03330; PACTIUS P‐2020‐578). All patients received oral and written information on the study and gave written consent before inclusion according to the guidelines of the Danish Ethics Committee.

### 2.2 Inclusion in the present biomarker study of CRP, IL‐6, and YKL‐40

From 1 July 2016 to 30 June 2019, 3968 patients were referred via the dedicated fast track pathway, of which 873 (22%) patients were included in the MICA study. The inclusion criteria were patients referred to the Diagnostic Outpatient Clinic, age older than 18 years. In all, 120 patients were excluded due to reasons shown in Figure 1B, resulting in a total of 753 patients in the present biomarker study. The reasons for the low inclusion rate were the following: (1) Few percent of the referred patients did not show up to their appointment or refused participation; and (2) Due to high workflow in the outpatient clinic, the doctors did not have time to ask the patients to participate in the study. Most of the patients were therefore only included in the study when medical students were employed to inform patients about the MICA study, collect the signed informed consent and draw the blood samples.

Time to cancer diagnosis, cancer‐specific mortality and death due to other causes were recorded until 20 October 2020.

### 2.3 Study objectives

The primary study objective was to investigate the utility of plasma CRP, IL‐6 and YKL‐40 concentrations alone and as a combined score to predict risk of a cancer diagnosis within 3 months from blood sample collection at the time of referral to the diagnostic cancer patient pathway at the Diagnostic Outpatient Clinic at Copenhagen University Hospital—Herlev and Gentofte, Denmark (baseline visit). The secondary study objectives were to investigate whether these biomarkers (alone or as a combined score) could predict a specific cancer diagnosis and prognosis during follow‐up.

### 2.4 Setting, source population and organisation of the diagnostic cancer patient pathway

In Denmark, all the citizens have free access to public healthcare and are registered with a local general practitioner as the primary healthcare contact. If the general practitioner finds serious nonspecific signs and symptoms of cancer and the patient does not fit into any of the established organ‐specific cancer patient pathways (e.g. haematological, breast, head and neck, reproductive system, brain, skin, lung, colorectal and upper gastrointestinal cancer patient pathways), the general practitioner refers the patient to the diagnostic cancer patient pathway.^33^ Patients can also be referred from private and hospital‐based physicians. The referring physicians will often order and evaluate blood and urine tests, abdominal ultrasound, chest x‐ray or endoscopies prior to the diagnostic referral. However, these tests are not a criterion for referral to the cancer patient pathway. Occasionally, the referral will be based on symptoms and medical history and/or findings from the physical examination only. The diagnostic workup flow is depictured in Figure 1A.

### 2.5 Data sources and covariates

All clinical data and results of the predefined blood tests were collected from patient records and saved in individual Case Report Forms. The included covariates were selected based on earlier studies reporting them to be associated with risk of cancer and/or mortality.^33^, ^34^, ^35^, ^36^, ^37^, ^38^, ^39^, ^40^, ^41^ Baseline clinical parameters and covariates are listed in Table S1. Symptoms at the time of referral were reported by patients at first visit with a physician in the diagnostic outpatient clinic. No predefined categories were used and symptoms collection were based on the referral and the physicians' anamnesis. High alcohol consumption was defined as alcohol intake above 7 (women) and 14 (men) drinks per week (1 drink ≈12 g of alcohol). Body mass index was calculated by measured weight in kilograms (at enrolment) divided by measured height in meters squared. We calculated Charlson comorbidity index (CCI) using journal information on pre‐existing conditions and ECOG performance status (PS). Other covariates collected during follow‐up were surgery (yes/no), presence of metastasis, tumour size and stage.

The date a cancer was diagnosed, and the type of cancer based on tissue biopsies in the clinical pathological information system (The Danish Patobank) were obtained from the patient records together with the results of radiological findings, including computed tomography scan (CT), positron emission tomography‐computed tomography (PET‐CT) or magnetic resonance imaging **(**MRI). A few patients (*n* = 13) did not have a tissue biopsy performed since they were terminally ill or refused further diagnostic evaluation.

Cancer types were divided into the following categories based on the primary origin: basal cell, biliary tract, bladder, breast, central nervous system, cervical, colorectal, haematological‐ (including leukaemia, lymphoma, multiple myeloma and myeloproliferative neoplasia), head and neck, gastric, kidney, liver, lung, malignant melanoma, Merkle cell, neuroendocrine, oesophageal, ovarian, pancreatic, penile, prostate, sarcoma, squamous‐cell, and testicular cancer or unknown primary tumour. Premalignant diagnoses were categorised into monoclonal gammopathy of undetermined significance (MGUS), intraductal papillary mucinous neoplasm (IPMN), superficial spreading melanoma in situ (SSM) and idiopathic hypereosinophilic syndrome (IHES). If a cancer was diagnosed, the American Joint Committee on cancer (AJCC) Tumour, Node, Metastasis (TNM) classification for solid cancers and Lugano classification for lymphomas were recorded.

Date of death and causes of death were collected from patient records and categorised into death due to a cancer, cardiovascular disease or other/unknown cause.

### 2.6 Blood sample collection

Blood samples were obtained while patients were being evaluated in the Diagnostic Outpatient Clinic at Herlev and Gentofte Hospital and before a diagnosis was finally made. Peripheral blood was collected in one K3EDTA tube (1 × 9 ml), two serum gel tubes (2 × 8 ml) and two PAXgene blood RNA tubes (2 × 2.5 ml) (Becton & Dickinson). Samples were processed according to the nationally approved standard operating procedure for blood collection and handling. Whole blood (1 × 1.5 ml) was aliquoted and stored at −80°C. Within 2 h, tubes were centrifuged at 2000*g* at 4°C for 10 min. After centrifugation, EDTA plasma, buffy coat and serum were aliquoted into two tubes with 2 ml plasma EDTA, one tube with 1.5 ml buffy coat (from the EDTA tubes) and 4 tubes with 2 ml serum. Buffy coat, EDTA plasma and serum were stored at −80°C. The 2.5 ml whole blood in two PAXgene Blood RNA tubes was collected and handled according to the manufacturer's instructions. PAXgene Blood RNA tubes were kept at room temperature for 2–72 h, then frozen at −20°C for 24–48 h and thereafter stored at −80°C.

### 2.7 Biochemical analysis

CRP was determined in fresh serum samples as a part of routine blood tests according to manufacturer's instructions, using a previously validated highly sensitive CRP immunoturbidimetric method on an automated analyser (Kit‐test SENTINEL CRP Ultra (UD), 11,508 UD‐2.0/022015/09/23). The measurement range is 0.3–640 mg/L, with an intra‐assay coefficient of variation (CV) of <3% and an inter‐assay CV of <15%. Elevated CRP was defined as >10 mg/L.

IL‐6 was determined in thawed serum samples in duplicate by a commercial two‐site, sandwich‐type enzyme‐linked immunosorbent assay (ELISA) (HS600B, R&D Systems). The detection limit was 0.01 ng/L. The intra‐assay CV was ≤8% and the inter‐assay CV was ≤11%. Patients were grouped as having low or high values, dichotomized using a cut‐off for IL‐6 of >4.92 ng/L, the 95th percentile in healthy blood donors.^42^

YKL‐40 was determined in thawed serum samples in duplicate by a commercial two‐site, sandwich‐type ELISA (Quidel Corporation). The detection limit was 10 μg/L. The intra‐assay CV was <5% and the inter‐assay CV was <6%. Elevated serum YKL‐40 was defined as higher than the age‐corrected 95th percentile.^43^

CA 19‐9 was determined in fresh serum samples using the Immulite 2000 GI‐MA assay (Siemens, Catalogue Number L2KG12), a solid‐phase, two‐site sequential chemiluminescent immunometric assay. Imprecision was monitored with two internal controls at 16 and 83 kU/L, with coefficients of variation of 8% and 9%. Accuracy was monitored within the standard UK NEQAS programme. Elevated CA 19‐9 was defined as >37 kU/L.

CEA was determined in fresh serum samples using an Atellica IM analyzer. The measurement range is 0.5–10,000 μg/L, with an intra‐assay CV of <5% and inter‐assay CV of <6%. Elevated CEA was defined as >5 μg/L.

CA‐125 was determined in fresh serum samples using an Atellica IM analyzer. The measurement range is 20–12,000 kU/L, with an inter‐assay CV of <4.1%. Elevated CA‐125 was defined as >35 kU/L.

PSA was determined in fresh serum samples using an Atellica IM analyzer. The measurement rage is 0.01–10,000 μg/L, with an inter‐assay CV of 6.5%. Elevated PSA was defined as >4 μg/L.

### 2.8 Statistical analysis

Results are reported in accordance with the REMARK (Reporting Recommendations for Tumour Marker Prognostic Studies) guidelines.^44^ Descriptive statistics were performed to describe demographics and baseline clinical characteristics for all included individuals. Exposure variables were CRP, IL‐6, YKL‐40, CEA, CA 19‐9, PSA, age, gender, PS and CCI and outcome variables were detection of cancer, type of cancer and death. Results are described as absolute numbers, percentages, median and ranges. Spearman's correlation coefficient rank test was used to examine the relationships between plasma concentrations of CRP, IL‐6 and YKL‐40. Established tumour markers (CEA, CA 19‐9, CA‐125, PSA) were also included. Differences in median between groups were calculated using the Mann–Whitney *U*‐test. The associations between biomarkers and the risk of cancer were analysed using logistic regression analysis, and CRP, IL‐6 and YKL‐40 levels, also including established tumour markers (CEA, CA 19‐9, CA‐125, PSA) were either applied in the model using the dichotomized values or as log2‐transformed continuous values, resulting in odds ratio (OR) per twofold increases in biomarker levels.

Discriminations between cancer and non‐cancer patients were evaluated by the receiver operator curve (ROC) and the area under the curve (AUC), with OR and 95% confidence intervals (CI) for each biomarker separately and for the number of elevated biomarkers.

The cumulative mortality in relation to plasma CRP, IL‐6 and YKL‐40, and CEA, CA 19‐9, CA‐125 and PSA levels (alone and combined) were plotted on Kaplan–Meier curves by dichotomizing the protein levels according to pre‐specified definitions. Inequality between groups was tested for by the log‐rank test. The relative risk of mortality was calculated by means of Cox proportional hazards regression for both continuous variables and dichotomized variables with time since baseline visit as the underlying time variable. This was presented as unadjusted hazard ratios (HRs) for two‐fold differences in biomarker levels and corresponding 95% CI as well as HRs adjusted for age, sex (when appropriate), PS and CCI. All analyses were either made in SAS version 9.4 or R Studio version 1.2 using a 5% significance level in two‐sided tests.

## 3 RESULTS

### 3.1 Patient characteristics

From July 2016 to July 2019, 873 patients at the Diagnostic Outpatient Clinic at Herlev and Gentofte Hospital, Copenhagen University Hospital, Denmark were included in the MICA study. We excluded 120 patients for the following reasons: duplicates (*n* = 5), missing or withdrew consent (*n* = 22), not enough blood for analysis (*n* = 85) and blood drawn after a cancer diagnosis was determined (*n* = 8) (Figure 1B). Of the remaining 753 patients, a total of 141 (19%) patients were diagnosed with cancer. Of these 141 patients with a cancer diagnosis, 111 (15%) were diagnosed with cancer within 3 months, and 30 patients (4%) were diagnosed in the remaining follow‐up period.

A total of 27 patients were diagnosed with basal‐cell carcinoma, 19 patients with the premalignant disease MGUS, t with IPMN, 3 with SSM and 1 with IHES. These 53 patients were added to the group of 559 participants not diagnosed with cancer during the follow‐up period and 612 patients were therefore assigned to the non‐cancer group (Figure 1B).

The clinical characteristics of the study cohorts are shown in Table 1. The most common referral symptoms were unexplained weight loss (52%) (mean weight loss 7.6 kg) and fatigue (37%). One hundred and thirty‐five (18%) patients had a previously diagnosed cancer, and 24 (18%) patients had active earlier cancer or signs of recurrence at time of inclusion.

**TABLE 1:** Baseline characteristics of the 753 included patients with suspicion of cancer

	No cancer diagnosis *N* = 612	Cancer diagnosis within 3 months *N* = 111	Cancer diagnosis after 3 months *N* = 30
Age, years, median (range)	69 (18–98)	75 (21–92)	73 (52–85)
Male	260 (42%)	56 (50%)	15 (50%)
Female	352 (58%)	55 (50%)	15 (50%)
Tobacco, current/former user	353 (60%)	66 (59%)	18 (64%)
Alcohol, current/former user	144 (26%)	29 (26%)	8 (31%)
BMI, underweight (<18.5)	40 (7%)	10 (9%)	2 (7%)
BMI, normal weight (18.5–25)	299 (49%)	51 (47%)	15 (52%)
BMI, overweight (25–30)	158 (26%)	37 (34%)	9 (31%)
Obesity (>30)	108 (18%)	11 (10%)	3 (10%)
PS 0	478 (78%)	71 (63%)	19 (66%)
PS 1	107 (17%)	27 (24%)	10 (34%)
PS ≥2	24 (4%)	14 (13%)	0 (0%)
CCI 0	322 (53%)	53 (47%)	9 (31%)
CCI 1–2	221 (36%)	40 (36%)	18 (62%)
CCI ≥3	69 (11%)	19 (17%)	2 (7%)
Diabetes	75 (12%)	15 (13%)	5 (17%)
Acute myocardial infarction	19 (3%)	2 (2%)	2 (7%)
Chronic heart failure	41 (7%)	14 (13%)	1 (3%)
Hypertension	126 (21%)	26 (23%)	4 (14%)
CEA, μg/L, median (range)	3 (1–18)	2 (1–1140)	2 (1–7)
CEA, % with elevated levels	5%	26%	10%
CA 19–9, kU/L, median (range)	9 (0.3–4440)	17 (0.3–33,200)	16 (0.3–140)
CA 19–9, % with elevated levels	11%	26%	24%
CA‐125, kU/L, median (range)	18 (0.2–1010)	12 (3–31,390)	10 (3–2290)
CA‐125, % with elevated levels	6%	15%	13%
PSA, μg/L, median (range)	1 (0.1–36)	2.8 (0.2–1794)	1.3 (0.1–7.6)
PSA, % with elevated levels	12%	35%	36%
CRP mg/L, median (range)	1.8 (0.1–273)	6.9 (0.1–131)	3.8 (0.5–56.5)
CRP, % with elevated levels	15%	44%	34%
IL‐6 ng/L, median (range)	2.9 (0.4–5000)	6.4 (0.4–925)	6.7 (1.1–19.2)
IL‐6, % with elevated levels	33%	60%	62%
YKL‐40 μg/L, median (range)	106 (20–4640)	176 (24–3035)	190 (31–1093)
YKL‐40, % with elevated levels	25%	45%	37%

*Note*: Values are number (%) if not otherwise stated.

Abbreviations: BMI, body mass index; CA 19‐9, carbohydrate antigen; CCI, Charlson comorbidity index; CEA, carcinoembryonic antigen; CRP, C‐reactive protein; DM, diabetes mellitus; IL‐6, interleukin 6; PS, performance status; PSA, prostate specific antigen.

The baseline clinical characteristics categorised by type of cancer diagnosed within 3 months is shown in Table S2.

### 3.2 Cancer events

A detailed outline of time from blood sampling until a cancer is diagnosed is shown in Figure 1C. Sixty‐six per cent of cancers were diagnosed within 1 month. The most common cancer diagnoses were lung cancer (*n* = 21, 15%), colorectal cancer (*n* = 16, 11%) and breast cancer (*n* = 11, 9%). Ten (7%) patients were diagnosed with a recurrence of earlier cancer. The number of each cancer type, the number and type of former cancers, and number of patients with signs of active earlier cancer are shown in Table S3.

### 3.3 Biomarkers in relation to cancer diagnosis

Among patients diagnosed with cancer within 3 months, 44% had elevated CRP, 60% had elevated IL‐6 and 45% had elevated YKL‐40. For the patients diagnosed with cancer during the remaining follow‐up period, 34% had elevated CRP, 62% had elevated IL‐6 and 37% had elevated YKL‐40 (Table 1). In patients not diagnosed with cancer, 15% had elevated CRP, 33% had elevated IL‐6 and 25% had elevated YKL‐40.

Boxplots for each biomarker in the different groups (non‐cancer group, cancer within 3 months and cancer during later follow‐up) are shown in Figure S1A–G. Patients diagnosed with cancer within 3 months had higher plasma CRP (*p* < 0.001), IL‐6 (*p* < 0.001), YKL‐40 (*p* < 0.001), CA 19‐9 (*p* < 0.001), CEA (*p* < 0.001), CA‐125 (*p* = 0.0075) and PSA (*p* < 0.001) than patients not diagnosed with cancer.

In patients diagnosed with cancer within 3 months, CRP correlated with IL‐6 (*r* = 0.75) and YKL‐40 (*r* = 0.42) and IL‐6 correlated with YKL‐40 (*r* = 0.46). In patients not diagnosed with cancer, CRP correlated with IL‐6 (*r* = 0.59) and YKL‐40 (*r* = 0.34), and IL‐6 with YKL‐40 (*r* = 0.54). In patients diagnosed with cancer, age was less correlated with the biomarkers (IL‐6 *r* = 0.31; YKL‐40 *r* = 0.24) than it was in patients not diagnosed with cancer (IL‐6 *r* = 0.36; YKL‐40 *r* = 0.42) (Table S4).

### 3.4 Diagnostic accuracy of the biomarkers for detection of cancer

**FIGURE 2:**
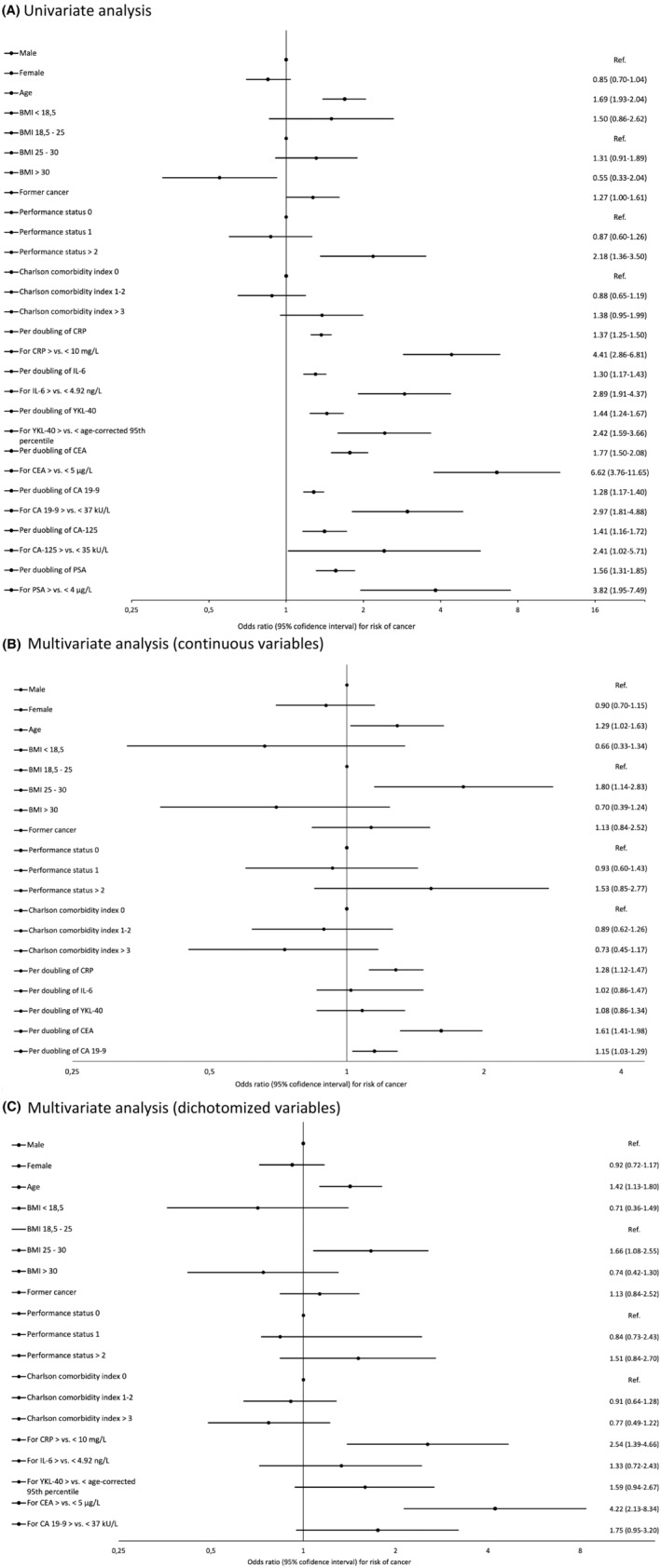
Graphical presentation of odds ratio (OR) and 95% confidence interval (CI) for univariable analysis (A), multivariate analysis using biomarkers as continuous values (B), and multivariate analysis using biomarkers as dichotomized values (C).

The unadjusted univariate analyses showed increased risk of cancer with increasing plasma CRP, IL‐6, YKL‐40, CEA, CA 19‐9, CA‐125, PSA, age, former cancer diagnosis and PS ≥2 (Figure 2A).

Table 2 shows the AUC, sensitivity (70%, 80% and 90%) and specificity for the biomarkers. Using a sensitivity of 90%, corresponding to a cut‐off for CRP > 16.5, the specificity of continuous CRP was 0.35, the positive predictive value was 0.39 and the negative predictive value was 0.88. Using a cut‐off for CRP > 10 mg/L, the sensitivity was 0.44, specificity was 0.85, the positive predictive value was 0.35 and the negative predictive value was 0.89.

**TABLE 2:** Sensitivity and specificity for the biomarkers to predict cancer

Marker cont.	AUC	Sensitivity at 70%				Sensitivity at 80%				Sensitivity at 90%			
		Cut‐off	Specificity	PPV	NPV	Cut‐off	Specificity	PPV	NPV	Cut‐off	Specificity	PPV	NPV
CRP	0.69	3.9	0.60	0.27	0.91	6.5	0.53	0.32	0.90	16.6	0.35	0.39	0.88
IL‐6	0.67	5.8	0.56	0.25	0.90	10.0	0.36	0.25	0.87	25.1	0.15	0.22	0.85
YKL‐40	0.66	161.0	0.56	0.25	0.90	227.0	0.41	0.27	0.88	441.0	0.19	0.26	0.86
CEA	0.64	3.0	0.47	0.24	0.88	4.0	0.40	0.33	0.89	5.0	0.32	0.44	0.88
CA 19–9	0.64	18.0	0.48	0.23	0.88	27.0	0.33	0.24	0.87	39.0	0.26	0.32	0.87
CA‐125	0.61	15.0	0.44	0.19	0.89	18.0	0.37	0.23	0.89	27.0	0.22	0.26	0.88
PSA	0.72	1.9	0.59	0.30	0.89	2.7	0.52	0.35	0.88	5.4	0.33	0.42	0.86

Marker dichotomized	AUC	Sensitivity	Specificity	PPV	NPV
CRP > 10 mg/L	0.65	0.44	0.85	0.35	0.89
IL‐6 > 4.92 ng/L	0.63	0.59	0.66	0.24	0.90
YKL‐40 > 95th percentile	0.60	0.45	0.75	0.24	0.88
CEA >5 μg/L	0.60	0.26	0.95	0.48	0.88
CA 19–9 > 37 kU/L	0.58	0.26	0.89	0.31	0.87
CA‐125 > 35 kU/L	0.54	0.15	0.93	0.26	0.86
PSA >4 μg/L	0.61	0.35	0.88	0.38	0.87

The univariate age‐ and sex‐adjusted model using log2‐transformed continuous variables showed that a doubling of CRP (OR = 1.30, 95% CI 1.18–1.43, *p* < 0.001), IL‐6 (OR = 1.20, 95% CI 1.07–1.34, *p* = 0.001) and YKL‐40 (OR = 1.28, 95% CI 1.09–1.51, *p* = 0.003) were associated with an increased risk of cancer.

When the biomarkers were used as dichotomized variables in the univariate age‐ and gender‐adjusted model, elevated CRP (>10 mg/L) (OR = 3.36, 95% CI 2.14–5.28, *p* < 0.001), IL‐6 (>4.92 ng/L) (OR = 2.18, 95% CI 1.42–3.36, *p* < 0.001) and YKL‐40 (>age‐corrected 95th percentile age‐corrected 95th percentile) (OR = 2.20, 95% CI 1.43–3.39, *p* < 0.001) were associated with an increased risk of cancer.

The ROC analysis of the non‐adjusted log2‐transformed variables showed that PSA (AUC = 0.72) and CRP (AUC = 0.69) had the highest AUC for the prediction of cancer. This was slightly lower for IL‐6 (AUC = 0.67) and YKL‐40 (AUC = 0.66) (Table 2). If CRP, IL‐6 and YKL‐40 were combined, the AUC was 0.71 for predicting cancer in the cohort of patients with non‐specific signs and symptoms of cancer. A combination of CRP, IL‐6, YKL‐40, CEA and CA 19‐9 was the best predictor of cancer (AUC of 0.77).

In multivariate analysis including continuous CRP, IL‐6, YKL‐40, CEA, CA 19‐9, age, sex (dichotomized), BMI (categorised), PS, CCI and a former cancer diagnosis (dichotomized), CRP (OR = 1.28, 95% CI 1.12–1.47, *p* < 0.001), CEA (OR = 1.61, 1.41–1.98, *p* < 0.001), CA 19‐9 (OR = 1.15, 1.03–1.29, *p* = 0.014), a 10‐year increase in age (OR = 1.29, 1.02–1.63, *p* = 0.037) and BMI 25–30 (OR = 1.80, 1.14–2.83, *p* = 0.010) were associated with a cancer diagnosis (Figure 2B).

In multivariate analysis including dichotomized CRP, IL‐6, YKL‐40, CEA, CA 19‐9, age (continuous), sex, BMI (categorised), PS, CCI and a former cancer diagnosis, CRP (OR = 2.54, 95% CI 1.39–4.66, *p* = 0.003), CEA (OR = 4.22, 2.13–8.34, *p* < 0.001), a 10‐year increase in age (OR = 1.42, 1.13–1.80, *p* = 0.003) and BMI 25–30 (OR = 1.66, 1.08–2.55, *p* = 0.022) were associated with a cancer diagnosis (Figure 2C).

### 3.5 CRP, IL‐6, YKL‐40 and prediction of overall survival

**FIGURE 3:**
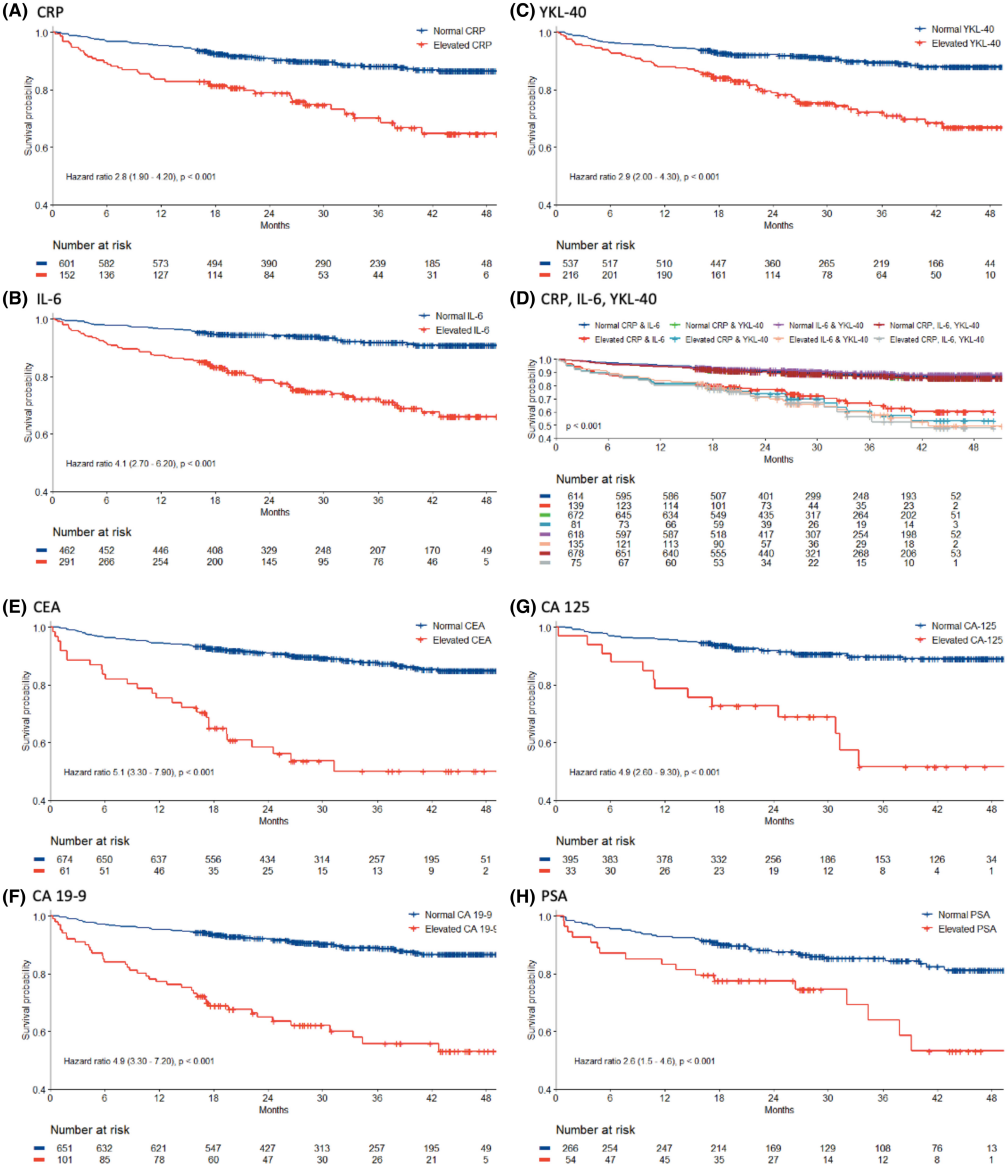
Kaplan–Meier curves in all patients showing overall survival (OS) according to high versus. normal levels of CRP (A), IL‐6 (B), YKL‐40 (C), combinations of CRP, IL‐6, YKL‐40 (D), CEA (E), CA 19–9 (F), CA‐125 (G), and PSA (H).

A total of 106 of the 753 included patients (14%) died within the follow‐up period. The cancer‐specific mortality was 7.4% (56 patients). In the 141 patients diagnosed with cancer, the overall mortality was 47% (66 patients) during the follow‐up period and the cancer‐specific mortality was 39% (55 patients). In the 612 patients not diagnosed with cancer, the overall mortality was 6% (37 patients). Patients referred to the diagnostic unit with elevated CRP, IL‐6 or YKL‐40 had significantly (*p* < 0.001) shorter OS than patients with normal biomarker levels (Figure 3A–C). Patients with combined high levels of CRP, IL‐6 and YKL‐40 had the shortest OS (HR = 3.8, 95% CI 2.5–5.9, *p* < 0.001) (Figure 3D). Patients with only elevated CEA, CA 19‐9, CA‐125 or PSA had significantly (*p* < 0.001) shorter OS than patients with normal biomarker levels (Figure 3E–H).

Patients referred to the diagnostic unit, with a cancer diagnosis, with only elevated IL‐6, CEA, CA 19‐9 or CA‐125 had significantly shorter OS than patients with a cancer diagnosis, without elevated biomarkers (Figure S2).

In all 753 patients, multivariate analysis including age, sex, PS, CCI and dichotomized CRP, IL‐6, YKL‐40, CEA and CA 19‐9 showed that elevated IL‐6 (HR = 1.88, 95% CI 1.14–3.1, *p* = 0.013), CEA (HR = 2.54, 1.58–4.1, *p* < 0.001), CA 19‐9 (HR = 2.72, 1.77–4.2, *p* < 0.001) and a 10‐year increase in age (HR = 1.68, 1.34–2.1, *p* < 0.001) were prognostic for OS. Analysis of continuous biomarker variables showed that YKL‐40 (HR = 1.23, 1.04–1.40, *p* = 0.015), CEA (HR = 1.18, 1.06–1.3, *p* = 0.003), CA 19‐9 (HR = 1.23, 1.13–1.3, *p* < 0.001), a 10‐year increase in age (HR = 1.54, 1.23–1.3, *p* < 0.001) and a PS of ≥2 (HR = 2.01, 1.01–4.0, *p* = 0.046) were prognostic for OS (Table S4).

## 4 DISCUSSION

In 2012, the Danish Health Authority implemented a fast‐track cancer patient pathway designed for patients with nonspecific signs and symptoms of cancer. The purpose was to reduce delays in the diagnosis of cancer and thereby enhance survival probability.^33^, ^34^ In the Diagnostic Outpatient Clinic, a panel of routine blood tests was collected as part of initial screening and additional tests could be ordered during the diagnostic work‐up.

Inflammation is one of the hallmarks of cancer and plays an important role in cancer development and progression.^10^ Plasma concentrations of the inflammatory biomarkers CRP, IL‐6 and YKL‐40 are associated with poor prognosis in patients with advanced cancer.^23^, ^24^, ^25^, ^26^, ^27^, ^32^ In this prospective biomarker study of 753 patients referred to a Diagnostic Outpatient Clinic in Denmark, 111 patients (15%) were diagnosed with cancer within 3 months. The diagnostic performance was best for plasma CRP compared to the other two inflammatory biomarkers. If the general cut‐off for CRP (>10 mg/L) was used, the sensitivity was 0.44, specificity was 0.85, the positive predictive value was 0.35 and the negative predictive value was 0.89 for a cancer diagnosis. The multivariate analysis also showed that elevated CRP was associated with an increased risk of cancer. A previous study of patients referred to another Diagnostic Outpatient Clinic in Denmark also found that high CRP and soluble urokinase plasminogen activator receptor were associated with cancer diagnosis.^41^ No earlier studies have evaluated the diagnostic utility of plasma IL‐6 and YKL‐40 in patients referred to a Diagnostic Outpatient Clinic with nonspecific signs and symptoms of cancer (Table S6).^33^, ^34^, ^35^, ^36^, ^37^, ^38^, ^39^, ^40^, ^41^, ^45^, ^46^

Therefore, CRP alone cannot identify patients with cancer, but when elevated, it may be used to indicate whether further diagnostic evaluation is needed in patients with nonspecific signs and symptoms of cancer. We hope that there will be a general awareness of suspicion of cancer in a subject with nonspecific symptoms of cancer and with high plasma CRP and no known inflammatory disease.

In accordance with earlier studies of patients with cancer,^23^, ^24^, ^25^, ^26^, ^27^, ^28^, ^29^, ^30^, ^31^, ^32^, ^35^, ^41^ we found that high plasma concentrations of CRP, IL‐6 and YKL‐40 either alone or combined were associated with short OS in patients suspected of cancer. This was also found for CEA, CA 19‐9, PSA and CA‐125.

A limitation of our study was that the sample size of patients with the different types of cancer was small and too low to determine whether specific cancer types had higher levels of inflammatory biomarkers, and whether the diagnostic and prognostic utility of the biomarkers were different between the cancer types. Therefore, the study was not able to provide insight regarding the diagnostic and prognostic utility of the biomarkers tested regarding specific types of cancer.

A strength of our study is the large group of patients with nonspecific signs and symptoms of cancer referred to a Diagnostic Unit. But a limitation of this explorative study of the diagnostic utility of plasma CRP, IL‐6 and YKL‐40 in detection of cancer is the lack of a validation cohort. A much larger study, with longer follow‐up time, is needed to better determine the risk of false negatives, false positives and overdiagnosis. The potential consequences should also be studied at the societal and the patient level if plasma CRP is included as a routine biomarker in patients referred with nonspecific signs and symptoms of cancer to a Diagnostic Unit. We have recently started to include more patients in the MICA study (up to 3000) for this purpose.

In conclusion, our study showed that plasma CRP, IL‐6 and YKL‐40 alone or combined cannot be used to identify patients with cancer. However, it did indicate that a combination of CRP, IL‐6, YKL‐40, CEA and CA 19‐9 can identify a subgroup of patients with nonspecific signs and symptoms of cancer who will develop cancer, and that they have a very poor prognosis if all biomarkers are elevated. Further studies are needed before the low‐cost biomarker CRP can be recommended to be used to indicate whether further diagnostic evaluation is needed when patients present with nonspecific signs and symptoms of cancer.

## AUTHOR CONTRIBUTIONS

**Alex Neergaard Videmark:** Data curation (lead); formal analysis (supporting); writing – original draft (lead); writing – review and editing (equal). **Ib J Christensen:** Formal analysis (lead). **Claus Larsen Feltoft:** Conceptualization (equal); funding acquisition (equal); writing – review and editing (equal). **Mette Jegstrup Villadsen:** Data curation (supporting); writing – review and editing (equal). **Frederikke Borg:** Data curation (equal). **Barbara Meyer Jørgensen:** Data curation (equal). **Stig E Bojesen:** Conceptualization (equal); writing – review and editing (equal). **Caroline Michaela Kistorp:** Conceptualization (equal); writing – review and editing (equal). **Randi Kjaersgaard Ugleholdt:** Conceptualization (equal); data curation (equal); writing – review and editing (lead). **Julia S. Johansen:** Conceptualization (lead); data curation (equal); funding acquisition (equal); writing – review and editing (lead).

## FUNDING INFORMATION

The salaries of ANV, FH and BMJ and analysis of CRP, IL‐6 and YKL‐40 were paid by the Departments of Medicine and Oncology, Herlev and Gentofte Hospital, Denmark.

## CONFLICT OF INTEREST

The authors declare no conflict of interest.

## CONSENT FOR PUBLICATION

Obtained from all authors.

## Supporting information


Data S1

## Data Availability

The dataset generated during the current study is available after approval from the study group and the Danish Data Protection Agency.
