# Supplementary material for: Combined plasma C‐reactive protein, interleukin 6 and YKL‐40 for detection of cancer and prognosis in patients with serious nonspecific symptoms and signs of cancer
Source: Cancer Med. 2022 Nov 28;12(6):6675–88. doi: 10.1002/cam4.5455 (PMC10067028; doi:10.1002/cam4.5455)
Supplement: Supplementary file 1 — Data S1 [file CAM4-12-6675-s001.docx]

**Supplementary Figure 1.**


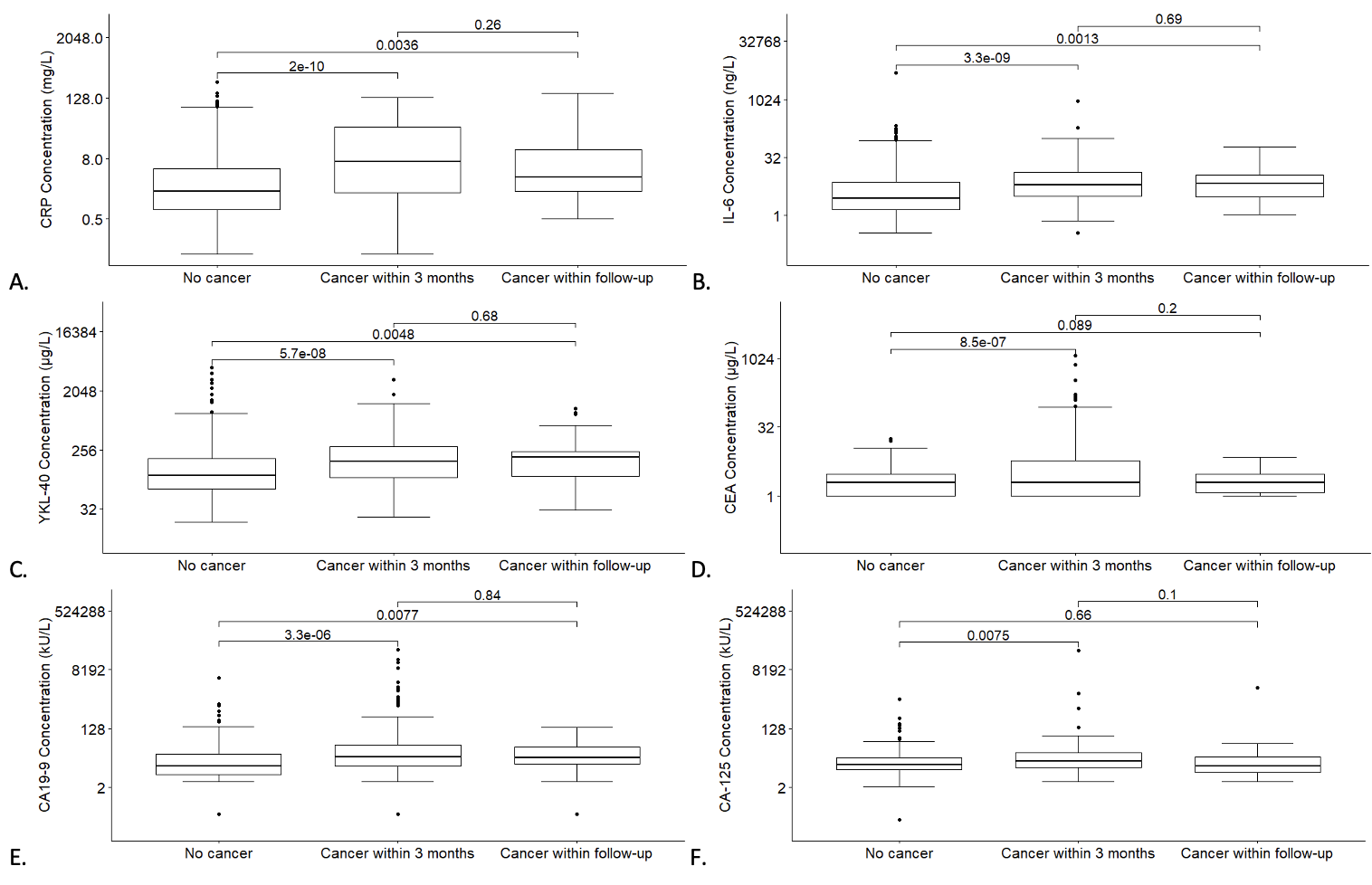


G.
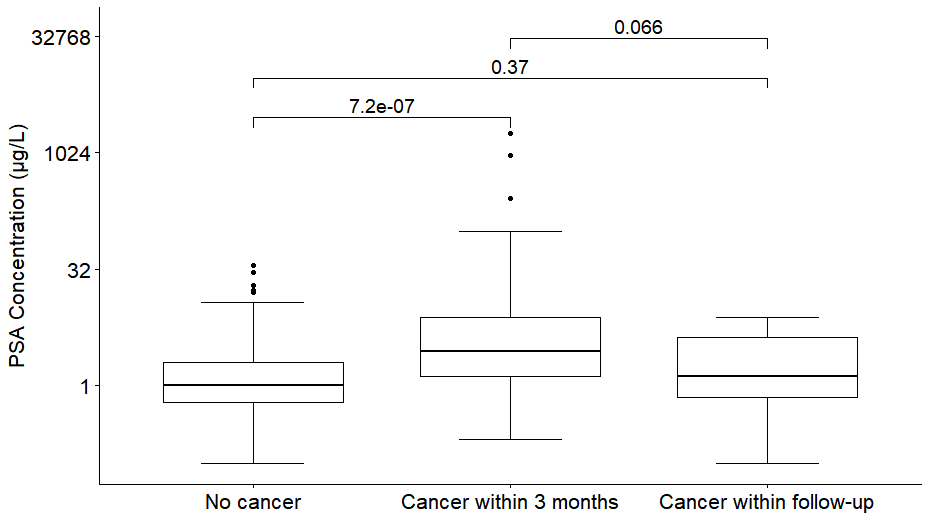


**Supplementary figure 2.** Kaplan-Meier curves showing overall survival (OS) in patients with a cancer diagnosis.

**
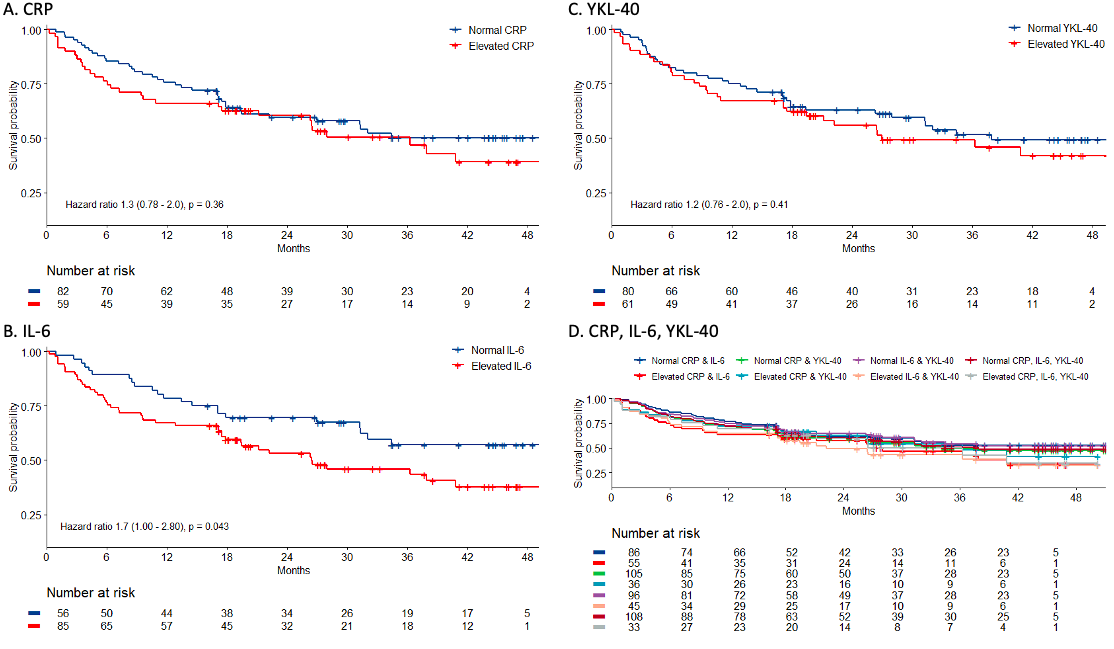
**

**Supplementary figure 2.** Kaplan-Meier curves showing overall survival (OS) in patients with a cancer diagnosis.

**
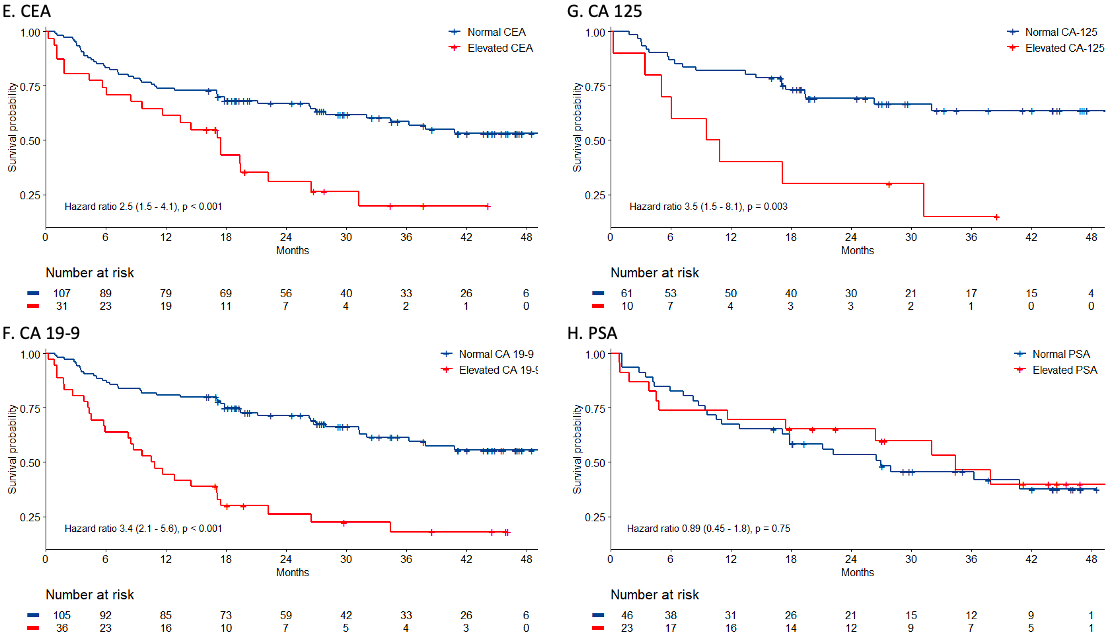
**

## Supplemental Table 1. Covariates

| **Clinical** **parameters** |
| --- |
| Sex |
| Age |
| Referring physician |
| Physician in the diagnostic outpatient clinic |
| Smoking status |
| Alcohol intake |
| Earlier cancer, including type and stage |
| **Symptoms** |
| General malaise |
| Fatigue |
| Non-intended weight loss |
| Night sweats |
| Diffuse pain |
| Reduced appetite |
| Fever |
| Enlarged lymph nodes |
| Noncharacteristic abdominal symptoms |
| Other symptoms |
| **Blood test including routine blood test for organ function** |
| C-reactive protein (CRP) |
| Carbohydrate antigen (CA 19-9) |
| Carcinoembryonic antigen (CEA) |
| Cancer antigen 125 (CA-125) |
| Prostate specific antigen (PSA) |
| **Comorbidities (Charlson comorbidity index)** |
| Cancer within the last 5 years (ex. non-melanoma skin cancer) |
| Cerebrovascular disease |
| Chronic pulmonary disease |
| Congestive heart failure |
| Dementia |
| Diabetes, with and without chronic complications |
| Hemiplegia |
| HIV, AIDS |
| Lymphoma/leukaemia within the last 5 years |
| Metastatic solid tumours |
| Mild liver disease, moderate or severe liver disease |
| Myocardial infarction |
| Peptic ulcer disease |
| Peripheral vascular disease |
| Renal disease |
| Rheumatic disease |

**Supplemental Table 2.** Characteristics of patients diagnosed with cancer within 3 months.

| **Cancer diagnosis within 3 months** | **N** | **Classification of cancer disease†** | | | | **Earlier cancer** | **Male**  **(%)** | **Female**  **(%)** | **Age***  **Years** | **PS**  **0/1/≥2** | **CCI*** |
| --- | --- | --- | --- | --- | --- | --- | --- | --- | --- | --- | --- |
|  |  | **1** | **2** | **3** | **4** |  |  |  |  |  |  |
| **B-cell lymphoma** | 5 | 2 | 0 | 0 | 3 | 2 | 60 | 40 | 79 (58-93) | 3/1/1 | 1.4 (0-2) |
| **Biliary tract cancer** | 6 | 1 | 0 | 1 | 7 | 2 | 50 | 50 | 74 (46-85) | 3/1/2 | 0.8 (0-2) |
| **Breast cancer‡** | 10 | 1 | 0 | 1 | 8 | 6 | 0 | 100 | 68 (21-85) | 7/1/2 | 0.4 (0-2) |
| **Colorectal cancer‡** | 15 | 4 | 2 | 3 | 6 | 4 | 60 | 40 | 76 (55-85) | 9/4/2 | 1.4 (0-4) |
| **DLBCL** | 3 | 0 | 1 | 0 | 2 | 0 | 100 | 0 | 79 (76-82) | 1/2/0 | 0.7 (0-2) |
| **Kidney cancer ‡** | 4 | 2 | 0 | 1 | 1 | 1 | 50 | 50 | 77 (70-90) | 4/0/0 | 1.25 (0-3) |
| **Liver cancer** | 3 | 1 | 0 | 1 | 1 | 1 | 33 | 67 | 64 (59-74) | 1/1/1 | 1.0 (0-2) |
| **Lung cancer** | 15 | 1 | 1 | 4 | 9 | 4 | 27 | 73 | 73 (62-82) | 11/3/1 | 1.1 (0-8) |
| **Multiple Myeloma** | 8 | - | - | - | - | 0 | 37 | 63 | 68 (54-80) | 4/4/0 | 0.6 (0-4) |
| **Other cancer** | 12 | 2 | 1 | 0 | 6 | 1 | 50 | 50 | 72 (45-92) | 11/1/0 | 0.64 (0-3) |
| **Other haematological** | 9 | 0 | 0 | 1 | 0 | 2 | 6 | 3 | 75 (67-84) | 4/4/1 | 2 (0-4) |
| **Pancreas cancer** | 5 | 0 | 1 | 2 | 2 | 1 | 60 | 40 | 86 (81-89) | 4/1/0 | 3.8 (2-6) |
| **Prostate cancer** | 9 | 1 | 0 | 1 | 7 | 2 | 100 | 0 | 70 (63-83) | 7/2/0 | 0.9 (0-4) |
| **UPT** | 7 | 0 | 0 | 0 | 1 | 1 | 57 | 43 | 75 (64-82) | 2/1/4 | 2.4 (1-7) |

* Values are mean (range).

Abbreviations: CCI, Charlson comorbidity index; DLBCL, Diffuse large B-cell lymphoma; PS, performance status; UPT, Unknown Primary Tumour.

Other: Connective tissue tumor (n=2), ependymom intraspinal tumor (n=1), esophagus cancer (n=2), gatric cancer (n=1), gastrointestinal stromal tumour (GIST) (n=1), glioblastoma (n=1), neuroendocrine tumor (n=2), thyroid cancer (n=1), and uterus cancer (n=1).

Other haematological: Angioimmunoblastic T-cell lymphoma (n=1), CLL (n=1), CML (n=1), essential thrombocytose (n=2), extraosseus plasmacytoma (n=1), MDS (n=2), and T-cell large granular lymphocytic leukemia (n=1).

† Either classified according to AJCC 8^th^ edition or Lugano classification for lymphomas. If neither were applicable, N/A was used.

‡ Cancers with recurrence: breast cancer (n=5); kidney cancer (n=2); and rectal cancer (n=2).

**Supplemental Table 3.** Individual cancer diagnoses within 3 months and during follow-up.

|  | **Cancer within 3 months (*n* = 111*)** | | | **Cancer within follow-up (*n* = 30*)** | | |
| --- | --- | --- | --- | --- | --- | --- |
|  | **N** | **Former cancer**  **N and type** | **Signs of earlier cancer** | **N** | **Type of former cancer** | **Signs of earlier cancer** |
| **Acute myeloid leukaemia** | 0 | 0 | 0 | 1 |  |  |
| **Angio immunoblastic T cell lymphoma** | 1 | 0 | 0 | 0 |  |  |
| **B-cell lymphoma** | 5 | 2 prostate cancer | 2 | 0 |  |  |
| **Biliary tract cancer** | 6 | 2 breast cancer | 0 | 1 |  |  |
| **Bladder cancer** | 0 |  |  | 1 |  |  |
| **Breast cancer** | 10 | 5 breast cancer,  1 sarcoma, 1 lymphoma | 4 | 1 | 1 SCC | 0 |
| **Caecum cancer** | 5 |  |  | 0 |  |  |
| **Chronic lymphatic leukaemia** | 1 |  |  | 0 |  |  |
| **Chronic myeloid leukaemia** | 1 |  |  | 0 |  |  |
| **Colon cancer** | 8 | 1 lung and 1 prostate cancer | 1 | 0 |  |  |
| **Connective tissue cancer** | 2 |  |  | 0 |  |  |
| **Diffuse large B-cell lymphoma** | 3 |  |  | 0 |  |  |
| **Essential thrombocytosis** | 2 |  |  | 0 |  |  |
| **Extraosseous plasmacytoma** | 1 |  |  | 0 |  |  |
| **Follicular lymphoma** | 0 |  |  | 1 | 1 prostate cancer, 1 MM | 0 |
| **Gastric cancer** | 1 |  |  | 0 |  |  |
| **Gastrointestinal stromal tumour** | 1 |  |  | 0 |  |  |
| **Glioblastoma** | 1 | 1 colon cancer | 0 | 0 |  |  |
| **Intraspinal ependymoma** | 1 |  |  | 0 |  |  |
| **Kidney cancer** | 4 | 1 kidney cancer | 1 | 0 |  |  |
| **Liver cancer** | 3 | 1 breast cancer | 0 | 1 |  |  |
| **Lung cancer** | 15 | 1 CLL, 1 breast and 1 ovarian cancer | 1 | 6 | 1 caecum and 1 lung cancer | 0 |
| **Malignant melanoma** | 0 |  |  | 2 |  |  |
| **Merkel cell carcinoma** | 0 |  |  | 1 | 1 kidney cancer | 1 |
| **Multiple Myeloma** | 8 |  |  | 1 |  |  |
| **Myelodysplastic syndrome** | 2 | 1 breast cancer, 1 MDS | 1 | 1 |  |  |
| **Neuroendocrine tumour** | 2 |  |  | 2 |  |  |
| **Oesophagus cancer** | 2 |  |  | 1 |  |  |
| **Pancreatic cancer** | 5 | 1 bladder cancer | 0 | 2 |  |  |
| **Prostate cancer** | 9 | 1 colon cancer, 1 polycythaemia vera | 1 | 1 |  |  |
| **Rectum** | 2 | 2 rectal cancer | 2 | 1 |  |  |
| **Squamous cell carcinoma** | 0 |  |  | 3 |  |  |
| **T-cell large granular lymphocytic leukaemia** | 1 |  |  | 0 |  |  |
| **Thyroid cancer** | 1 |  |  | 0 |  |  |
| **Tongue cancer** | 0 |  |  | 1 | 1 malignant melanoma | 0 |
| **Unknown primary tumour** | 7 | 1 malignant melanoma | 0 | 1 |  |  |
| **Uterus cancer** | 1 | 1 breast cancer | 0 | 0 |  |  |

## * 10 patients had more than one cancer diagnosed within follow-up period (Only first diagnosis shown).

## Supplemental Table 4. Spearman’s correlations coefficient analysis between inflammatory biomarkers and age in the groups with and without cancer.

|  | **No cancer diagnosis** | | | | | | | |
| --- | --- | --- | --- | --- | --- | --- | --- | --- |
|  | **Age** | **CRP** | **IL-6** | **YKL-40** | **CEA** | **CA19-9** | **CA125**‡ | **PSA**‡ |
| **Age** | 1 | 0.22*** | 0.37*** | 0.42*** | 0.19*** | 0.17*** | 0.03 | 0.27*** |
| **CRP** | 0.22*** | 1 | 0.58*** | 0.34*** | 0.05 | 0.06 | 0.15** | 0.08 |
| **IL-6** | 0.37*** | 0.58*** | 1 | 0.53*** | 0.14*** | 0.13*** | 0.15** | 0.01 |
| **YKL-40** | 0.42*** | 0.34*** | 0.53*** | 1 | 0.25*** | 0.22*** | 0.15** | 0.12 |
| **CEA** | 0.19*** | 0.05 | 0.14*** | 0.25*** | 1 | 0.20*** | 0.02 | -0.02 |
| **CA19-9** | 0.17*** | 0.06 | 0.13*** | 0.22*** | 0.20*** | 1 | 0.21*** | 0.15* |
| **CA125** | 0.03 | 0.15** | 0.15** | 0.15** | 0.02 | 0.21*** | 1 | - |
| **PSA** | 0.27*** | 0.08 | 0.01 | 0.12 | -0.02 | 0.15* | - | 1 |

*P<0.05. ** P<0.01. ***P<0.001.

‡: CA125 in females. PSA in males

| **Cancer diagnosis within 3 months** | | | | | | | | |
| --- | --- | --- | --- | --- | --- | --- | --- | --- |
|  | **Age** | **CRP** | **IL-6** | **YKL-40** | **CEA** | **CA19-9** | **CA125**‡ | **PSA**‡ |
| **Age** | 1 | 0.27** | 0.32** | 0.24* | 0.01 | 0.11 | -0.09 | 0.11 |
| **CRP** | 0.27** | 1 | 0.75*** | 0.42*** | 0.11 | 0.07 | 0.17 | 0.11 |
| **IL-6** | 0.32** | 0.75*** | 1 | 0.46*** | 0.21* | 0.12 | 0.24 | -0.01 |
| **YKL-40** | 0.24* | 0.42*** | 0.46*** | 1 | 0.17 | 0.24* | 0.31* | -0.20 |
| **CEA** | 0.007 | 0.11 | 0.21* | 0.17 | 1 | 0.52*** | 0.30* | 0.07 |
| **CA19-9** | 0.11 | 0.07 | 0.12 | 0.24* | 0.52*** | 1 | 0.08 | -0.16 |
| **CA125** | -0.09 | 0.17 | 0.24 | 0.31* | 0.30* | 0.08 | 1 | - |
| **PSA** | 0.11 | 0.11 | -0.01 | -0.20 | 0.07 | -0.16 | - | 1 |

*P<0.05. ** P<0.01. ***P<0.001.

‡: CA125 in females. PSA in males

## Supplemental Table 5. Multivariate cox analysis of overall survival in all patients.

|  | **All patients (*N* = 753 and 104 deaths)** | | | | | |
| --- | --- | --- | --- | --- | --- | --- |
|  | **Dichotomized variables model** | | | **Continuous variables model** | | |
|  | **HR** | **95% CI** | ***p*-value** | **HR** | **95% CI** | ***p*-value** |
| **Sex** |  |  |  |  |  |  |
| Male | Ref. |  |  | Ref. |  |  |
| Female | 0.63 | 0.45-1.0 | 0.069 | 0.70 | 0.46-1.0 | 0.08 |
| **Age10** | 1.68 | 1.34-2.1 | <0.001 | 1.54 | 1.23-1.3 | <0.001 |
| **CCI** |  |  |  |  |  |  |
| 0 | Ref. |  |  | Ref. |  |  |
| 1 | 1.32 | 0.84-2.1 | 0.23 | 1.18 | 0.74-1.9 | 0.49 |
| ≥2 | 1.02 | 0.55-1.9 | 0.94 | 0.86 | 0.46-1.6 | 0.64 |
| **PS** |  |  |  |  |  |  |
| 0 | Ref. |  |  | Ref. |  |  |
| 1 | 1.36 | 0.84-2.2 | 0.21 | 1.57 | 0.98-2.5 | 0.06 |
| ≥2 | 1.90 | 0.96-3.8 | 0.065 | 2.01 | 1.01-4.0 | 0.046 |
| **CRP** | 1.22 | 0.77-1.9 | 0.39 | 1.03 | 0.93-1.2 | 0.54 |
| **IL-6** | 1.88 | 1.14-3.1 | 0.013 | 1.11 | 0.97-1.3 | 0.14 |
| **YKL-40** | 1.36 | 0.86-2.1 | 0.19 | 1.23 | 1.04-1.4 | 0.015 |
| **CEA** | 2.54 | 1.58-4.1 | <0.001 | 1.18 | 1.06-1.3 | 0.003 |
| **CA19-9** | 2.72 | 1.77-4.2 | <0.001 | 1.23 | 1.13-1.3 | <0.001 |

HR, Hazard ratio; CI, confidence interval; CCI, Charlson comorbidity index; PS, performance status; Age10, 10-year increase in age.

## Supplemental Table 6. Previous studies of patients with serious non-specific signs and symptoms of cancer (NSSC) referred to Diagnostic Outpatients Clinics performed in Denmark.

| **Article** | **Design and aim of study** | **Setting and population** | **Main results** |
| --- | --- | --- | --- |
| **Bislev LS**  **Dan Med J 2015** | Retrospective cohort.  Investigating the prevalence of cancer in patients with NSSC. | Aarhus Hospital  *N* = 323 | 18% were diagnosed with cancer. |
| **Ingemann ML BMC Cancer 2015** | Cross-sectional study.  Describing characteristics of NSSC patients and probability of cancer in this population. | Aarhus and Silkeborg Hospital  Patients referred from GP  *N* = 1278 | 16.2% were diagnosed with cancer.  Most frequent were lung, colorectal, haematological and pancreas cancer. No single biomarker was associated with a cancer diagnosis. |
| **Jørgensen SF BMC Cancer 2017** | Retrospective cohort study. Describing population and cancer pathway, prevalence of cancer, mortality, and risk factors associated with cancer. | Nordsjaelland’s Hospital *N* = 825 | 16.7% were diagnosed with cancer.  Most frequent were haematological, lung, and gastrointestinal cancer.  1-year overall mortality was 44.2% in cancer patients and 3.3% in non-cancer patients. Anaemia, leucocytosis, thrombocytopenia, elevated LDH, and CRP were associated with a cancer diagnosis. |
| **Lebech AM Nucl Med 2017** | Randomized controlled trial. Investigating whether F-FDG PET/CT was superior to CT as initial radiology examination. | Hvidovre Hospital Patients were randomized 1:1 for PET/CT and CT.  *N* = 197 | 20% were diagnosed with cancer.  Most frequent were lung, prostate. and breast cancer.  PET/CT had higher specificity and sensitivity and were superior as initial radiology examination. |
| **Moeller M**  **BMJ Open 2019** | Cohort study.  Investigating the diagnostic relevance of contrast enhanced CT. | Roskilde Hospital  *N* = 529 | 20% were diagnosed with cancer.  The negative predictive value was 98% and positive predictive value was 63%. |
| **Moseholm E Cancer Epidemiol 2017** | Cohort study.  Describing characteristics of patients and estimating prevalence of cancer and other diagnoses. | Nationwide in Denmark. *N* = 23,934 | Most frequent were breast, haematological colorectal, and pancreas cancer and malignant melanoma. |
| **Næser E**  **BMC Cancer 2017** | Cohort study.  Investigating the diagnostic value of standardized blood samples. | Silkeborg Hospital *N* = 1499 | 12.2% had a cancer diagnosis. Highest probability of cancer was seen in patients with high hCG, M-protein, CA-125, bilirubin, and low IgA. |
| **Næser E**  **Cancer Epidemiol 2018** | Matched cohort study. Investigating mortality in patients referred to the NSSC-CPP. Investigating difference in mortality in cancer patients diagnosed through the NSSC-CPP and cancer patients diagnosed by other means. | Silkeborg Hospital  *N* =938  *N* = 1103 matched controls | 12.6% were diagnosed with cancer.  All-cause mortality was 28% for cancer patients. There was no difference in cancer mortality between patients diagnosed in the NSSC-CPP and patients diagnosed by other means. |
| **Næser E**  **Cancer Epidemiol 2017** | Cohort study.  Investigating the distribution of cancer and non-cancer and the diagnostic value of clinical characteristics. | Silkeborg Hospital  *N* = 938 | 12.6% were diagnosed with cancer.  Most frequent symptom were weight loss, lowered appetite, pain, general condition.  30% had abnormal clinical characteristics. Most frequent were abdominal or rectal findings or swollen lymph node.  Patients with a lump, a process in the abdomen, high LDH or abnormal abdominal ultrasound had greatest probability of cancer. |
| **Rasmussen LJH Int J Cancer 2017** | Sub-study to Lebech (Nucl Med) 2017.  Randomized controlled trial. Investigating the diagnostic value of various biochemical analysis. | Hvidovre Hospital  Patients were randomized (1:1) to PET/CT or CT. *N* = 197. | 20% were diagnosed with cancer.  Most frequent were lung, prostate, and breast cancer.  Patients diagnosed with cancer had lower albumin and haemoglobin, higher CRP, ESR, and suPAR. Former cancer, CRP, and suPAR were associated with a cancer diagnosis. |
| **Rasmussen LJH Clin Biochem 2020** | Prospective cohort study. Investigating whether low suPAR could identify disease-free patients. | Hvidovre Hospital  *N* = 1583 | 22% were diagnosed with cancer.  suPAR was significantly lower in patients not diagnosed with cancer or other disease. Conclusion was that suPAR could be a prognostic marker for adverse outcome. |

Abbreviations: CPP, Cancer patient pathway; ESR, erythrocyte sedimentation rate; F-FDG PET/CT, Flour-Deoxy-Glucose Positron emission tomography; GP, General practitioner; hCG, human choriogonadotropin hormone; LDH, lactate dehydrogenase; NSSC, Non-specific-signs and symptoms of cancer; suPAR, soluble urokinase plasminogen activator receptor.
